# PROPOSAL AND TEST-RETEST RELIABILITY OF A SCALE FOR CERVICAL, THORACIC, AND LUMBAR SPINE PAIN IN BRAZILIAN YOUNG PEOPLE

**DOI:** 10.1590/1984-0462/;2019;37;4;00001

**Published:** 2019-01-07

**Authors:** Gustavo Aires de Arruda, Diogo Henrique Constantino Coledam, Arli Ramos de Oliveira, Fernanda dos Santos Neri, João Paulo de Aguiar Greca, Jefferson Rosa Cardoso

**Affiliations:** a Universidade Estadual de Londrina, Londrina, PR, Brasil.; b Instituto Federal de Educação, Ciência e Tecnologia de São Paulo, Boituva, SP, Brasil.; c Brunel University London, Uxbridge, Grande Londres, Inglaterra.

**Keywords:** Adolescent, Child, Neck pain, Low back pain, Pain measurement, Adolescente, Criança, Cervicalgia, Dor lombar, Medição da dor

## Abstract

**Objective:**

To propose and analyze the test-retest reliability of an instrument to verify the presence and intensity of pain in the cervical, thoracic and lumbar spine in Brazilian young people.

**Methods:**

This reliability study enrolled a sample of 458 participants (13 to 20 years). Two groups were formed for each sex according to the range of days for the test-retest (10±3 and 28±2 days). For analysis of spinal pain, a drawing of the human body with cervical, thoracic and lumbar spine areas delimited was presented. The following question was presented: during a normal day, do you feel pain in any of these regions of your spine? If so, what is the intensity from 0 to 10 (mark on the line)? The starting point, with the number 0, corresponded to no pain, and the number 10 to severe pain. The agreement of frequency and of intensity of pain was verified by *Kappa* test and Bland-Altman plot, respectively.

**Results:**

Intraclass correlation coefficients ranged from 0.71 (confidence interval of 95% - 95%CI - 0.59-0.79) to 0.94 (95%CI 0.90-0.96). The results concerning the agreement of pain scores showed the mean differences to be close to 0, and the largest mean difference was -0.40 (95%CI -5.14-4.34). The agreement in reported pain ranged from 72.2 (*Kappa* 0.43; 95%CI 0.28-0.58) to 90.1% (*Kappa* 0.76; 95%CI 0.60-0.92).

**Conclusions:**

This instrument was shown to be a reliable manner to verify the pain in different regions of the spine in Brazilian young people.

## RESUMO

**Objetivo:**

Propor e analisar a reprodutibilidade de um instrumento para verificar a presença e a intensidade da dor na coluna cervical, torácica e lombar em jovens brasileiros.

**Métodos:**

Estudo de reprodutibilidade com uma amostra de 458 participantes (13 a 20 anos). Dois grupos foram formados para cada sexo de acordo com o intervalo de dias entre teste e reteste (10±3 e 28±2 dias). Para a análise da dor na coluna, foi apresentada a figura de um corpo humano com as áreas da coluna cervical, torácica e lombar delimitadas. A seguinte pergunta foi realizada: durante um dia comum, você sente dor em alguma dessas regiões da coluna? Se sim, qual é a intensidade de 0 a 10 (marque um traço)? A extremidade com o número 0 correspondia à ausência de dor e o número 10, à dor muito intensa. A concordância na frequência e intensidade da dor foi verificada por meio do teste *Kappa* e da plotagem de Bland-Altman, respectivamente.

**Resultados:**

Os coeficientes de correlação intraclasse variaram de 0,71 (intervalo de confiança de 95% - IC95% - 0,59-0,79) a 0,94 (IC95% 0,90-0,96). Os resultados relativos à concordância no escore de dor mostraram que as diferenças médias foram próximas de 0 e a maior diferença média foi de -0,40 (IC95% -5,14-4,34). A concordância no relato de dor variou de 72,2 (*Kappa* 0,43; IC95% 0,28-0,58) a 90,1% (*Kappa* 0,76; IC95% 0,60-0,92).

**Conclusões:**

O instrumento demonstrou ser uma forma reprodutível de verificar a dor em diferentes regiões da coluna vertebral em jovens brasileiros.

## INTRODUCTION

Among the regions in the human body affected by musculoskeletal pain, the lumbar spine has been widely investigated. A systematic review observed prevalence of low back pain varying from 9 to 65% in young people from different regions of the world.^1^ Recent studies described that the prevalence of low back pain in Brazilian children and adolescents ranges from 15.5 to 18%.^2^ ^,^ ^3^ ^,^ ^4^ The high prevalence of back pain can be considered a public health problem.^5^ In addition to low back pain, there is high prevalence (>20%) of young people that report pain on cervical and thoracic spine regions.^3^ Multiple pain sites are associated to disabilities in adolescents,^6^ and concomitant neck and low back pain increases the risk of mental health problems when compared to single pain.^7^ Early prevention is recommended, as low back pain in adolescence can predict low back pain in adulthood.^8^

Questionnaires are extensively used to assess back pain in children and adolescents. In Brazil, studies that aimed to assess back pain in children and adolescents failed to report the process of translation and cross-culturally adaptation^2^ ^,^ ^9^ and did not report its reproducibility data in Brazilian young people.^2^ ^,^ ^3^ ^,^ ^4^ ^,^ ^9^ ^,^ ^10^ ^,^ ^11^ Another usual limitation of questionnaires used in Brazilian studies is that pain is analyzed as a dichotomous way, *i.e.*, presence or absence of pain. Therefore, the intensity of pain cannot be estimated.^3^ ^,^ ^4^ ^,^ ^11^

The visual analogue scale is an instrument that can overcome the issue of dichotomous pain reports. This scale is frequently used to evaluate the intensity of the pain and has been largely used as a reference procedure in the validation of instruments for pain verification.^12^ ^,^ ^13^ ^,^ ^14^ Noll et al. proposed a Brazilian scale to assess back pain that can point out information regarding intensity, but it does not discriminate cervical, thoracic or low back pain.^15^

Low back pain assessment is necessary, as it has a complex etiology and can emerge from many causes,^5^ and the estimated total health cost of people living with chronic back pain seems to be doubled, when compared to those who do not mention any pain.^16^ However, high prevalence of pain in other regions of spine may affect children and adolescents. A Danish study described that neck pain was the most common spinal pain region, followed by mid back and low back pain.^17^ Still, consequences of multiple pain sites are not clear, as this issue has received sparse attention.

An instrument to analyze the frequency and intensity of pain in different regions of the spine would be relevant for professionals and researchers that need to identify the prevalence and to check the efficacy of intervention programs that aim to prevent or reduce cervical, thoracic and low back pain in children and adolescents. Therefore, the purpose of this study was to analyze the test-retest reliability of an instrument to verify the presence and intensity of pain in the cervical, thoracic and lumbar spine in Brazilian young people.

## METHOD

This is a reliability study that was part of a larger project that involved children and adolescents from Londrina, Paraná, Brazil. The larger project included information about physical activity, eating habits and consumption of alcoholic beverages, smoking, spinal pain, socioeconomic and demographic information by questionnaires, after that anthropometric measures, blood pressure and heart rate were collected, and it was performed the Fitnessgram motor tests. Study protocols were approved by the Ethics in Research Committee from the university where the study took place (Protocol no. 234/10). Parents or guardians of students who agreed to participate in the study signed a consent form wherein all the procedures, researcher contact details, and possible risks and benefits of the study were described.

Londrina city had 48,688 students enrolled in state schools (publicly administered institutions) at the beginning of the study, from 5^th^ grade of elementary school to the 3^rd^ grade of high school. The total of 30,777 students were attending the 5^th^ to 8^th^ grades. Regarding the 1^st^, 2^nd^ and 3^rd^ high school years, 17,911 students were enrolled in state schools (data from the City Department of Education of Londrina, referring to the year 2009). In the present study, the schools with 400 to 800 enrolled students were considered medium-sized schools, and the schools with more than 800 enrolled students were considered large. The number of enrolments was proportionally distributed among small, medium and large schools in the city. Two state schools in the city of Londrina were randomly selected for the composition of the sample in the present study: a medium-sized (central region) and a large one (northern region). Classrooms were randomly selected in each school (conglomerate). The sample involved approximately 50% of the participants of each school. Participants in this study were composed of 458 people (236 girls and 222 boys), in the age range from 13 to 20 years old.

Two groups were formed for each sex, according to the days for the test-retest. The first group consisted of 80 boys with the mean interval of test-retest of the pain scale 10±3 days, and the second group was composed of 142 boys and the mean interval between test-retest was 28±2 days. The same procedure was adopted for girls - the first group including 89 girls and the second group 147. A sample size of 80 participants with two observations achieved the power of 94.5%, considering as an alternative hypothesis the intraclass correlation coefficients (ICC) value of 0.70 and for the null hypothesis the value of 0.40, using the F-test with the significance level of 0.01. Under the same conditions, sample sizes of 89, 142 and 147 subjects had 96.5, 99.8 and 99.9% of power, respectively. All data was calculated using Power Analysis and Sample Size Software 15.

The following procedures were conducted to develop the instrument to evaluate back pain. First version of the instrument was developed, and its content was analyzed by four experts. Suggestions were examined and incorporated in a second version of the instrument, and experts then carried out a new content analysis. This version was used in young people to verify their understanding regarding the instrument and reproducibility.

**Figure 1:**
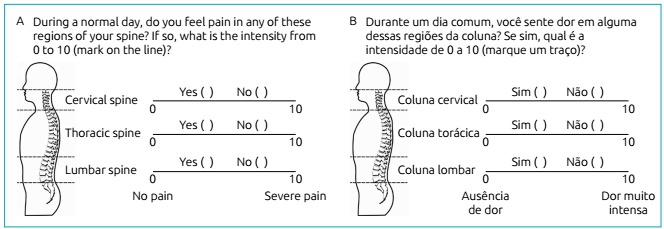
Scale for verifying the presence of spinal pain: (A) English version; (B) Portuguese version.

A drawing of the human body (lateral position), which made it possible to visualize the spine, was presented to the students to explore the presence of spinal pain (Figures 1A and 1B). The areas of the cervical, thoracic and lumbar spine were delimited by a dashed line and the name of the region indicated. The following question with the options *yes* or *no* was presented to students: during a normal day, do you feel pain in any of these regions of your spine? If so, what is the intensity from 0 to 10 (mark on the line)? The visual analog scale measured 10 cm. The starting point, with the number 0, corresponded to no pain; and the number 10, to severe pain.

The instrument was applied in the classroom during physical education classes. Only the students participating in the research remained in the room. Prior to responding the questionnaire, an explanation was given regarding the purpose of the instrument. While participants answered the questionnaire, possible doubts were explained. One of the researchers (GAA) was present during the whole procedures of data collection, and s/he received assistance from other researchers previously trained to perform the procedures in a standardized way. Participants were advised to disregard pains in other regions of the body other than the spine. Also, they were advised to ignore sporadic pain caused by recent trauma such as falls, knocks, etc., reporting only usual pain.

Additionally, in this study the translation of the instrument to English was carried out according to previous recommendations.^18^ Firstly, two professional translators translated the original Portuguese version to English (translations). During the translation process, equality of meaning was prioritized instead of equality of word. Subsequently, two Brazilian researchers in the field of health translated this version from English to Portuguese (back-translations). Finally, the research team reached a consensus regarding the final version of the instrument based on its first and second translation. This procedure was performed to facilitate the use of the instrument in other countries, thus making it possible to compare information about prevalence and intensity of spine pain in young people.

Normal distribution of the data was analyzed by the Kolmogorov-Smirnov’s test. Descriptive analyses used mean and standard deviation (SD). The Student’s unpaired t-test was performed to compare the characteristics between groups for boys, and equality of variances was averiguated by Levene’s test. The same tests were used to compare characteristics between girls. The test-retest reliability of pain scores was verified by the ICC one-way random effect and their respective 95% confidence intervals (95%CI). The interpretation was performed according to the values: <0.40 = poor; 0.40 to <0.75 = good; ≥0.75 = excellent.^19^ The agreement between the scores for test-retest was verified with the Bland-Altman plot method. The bias between the mean values of pain and interval of days (test-retest) was verified by regression models (linear, quadratic and cubic) and R-squared. The same procedure was used to check the bias between the mean values of pain and differences (test-retest). The agreement of reports for the presence of spinal pain according to the region was verified using the *Kappa* index, and the interpretation performed according to values: ≤0.20 = poor; 0.21 to 0.40 = regular; 0.41 to 0.60 = moderate; 0.61 to 0.80 = good; >0.80 = very good.^20^ The relative frequency and 95%CI of spinal pain according to the region were calculated. The comparisons of frequencies between test-retest for each group were performed using the McNemar’s test. Results were considered statistically significant when p≤0.05. All data were analyzed using *Statistical Package for the Social Sciences* (SPSS) 20.0.

## RESULTS

Table 1 presents the sample characteristics according to the gender and mean interval days (10 or 28 days) of spinal pain scale application. No differences were found between interval day groups in boys or girls (p>0.05). The mean age of all groups was 15 years old.

**Table 1:** Characteristics of the sample according to gender and interval of days between pain scale application.

	10±3 days	
	Boys (n=80) Mean±SD	Girls (n=89) Mean±SD
Age (years)	15.1±1.7	14.9±1.7
Body mass (kg)	63.5±16.0	52.9±11.8
Height (cm)	169.2±10.3	160.0±6.4
BMI (kg/m^2^)	22.1±4.7	20.6±3.9
	28±2 days	
	Boys (n=142) Mean±SD	Girls (n=147) Mean±SD
Age (years)	15.5±1.5	15.4±1.4
Body mass (kg)	61.9±12.3	54.7±11.1
Height (cm)	170.3±8.9	161.4±6.6
BMI (kg/m^2^)	21.2±3.1	20.9±3.9

BMI: body mass index; SD: standard deviation; p>0.05 for comparisons between groups of days for boys and girls.

Table 2 exhibits the ICC for the pain scale according to the spinal region. Table 3 contains the relative frequency of pain in the test and retest moments for each spinal region, while Table 4 shows the agreement in the indication of pain presence between test and retest instrument administration moments.

**Table 2:** Intraclass correlation coefficient and Bland-Altman plot for the pain scale, according to gender and interval of days between test-retest.

	Interval of 10±3 days			
	Boys (n=80)		Girls (n=89)	
	ICC (95%CI)	Bland-Altman (95%CI)	ICC (95%CI)	Bland-Altman (95%CI)
Cervical spine	0.94 (0.90-0.96)	-0.09 (-1.54-1.36)	0.82 (0.73-0.88)	-0.15 (-3.88-3.58)
Thoracic spine	0.85 (0.76-0.90)	0.02 (-2.65-2.69)	0.83 (0.74-0.89)	0.00 (-3.69-3.70)
Lumbar spine	0.77 (0.65-0.86)	-0.16 (-3.17-2.85)	0.92 (0.88-0.95)	0.05 (-3.07-3.17)
	Interval of 28±2 days			
	Boys (n=142)		Girls (n=147)	
	ICC (95%CI)	Bland-Altman (95%CI)	ICC (95%CI)	Bland-Altman (95%CI)
Cervical spine	0.71 (0.60-0.79)	0.19 (-3.19-3.57)	0.84 (0.77-0.88)	0.12 (-3.41-3.65)
Thoracic spine	0.84 (0.79-0.89)	-0.09 (-3.92-2.14)	0.71 (0.59-0.79)	-0.17 (-4.97-4.62)
Lumbar spine	0.79 (0.71-0.85)	0.15 (-3.61-3.92)	0.75 (0.65-0.82)	-0.40 (-5.14-4.34)

p<0.01 for all intraclass correlation coefficient values; 95%CI: 95% confidence interval.

**Table 3:** Relative frequencies of spine pain according to gender and interval of days between test-retest for the pain scale.

	Interval of 10±3 days			
	Boys (n=80)		Girls (n=89)	
	Test % (95%CI)	Retest % (95%CI)	Test % (95%CI)	Retest % (95%CI)
Cervical spine	26.3 (16.6-35.9)	31.3 (21.1-41.4)	39.3 (29.2-49.5)	43.8 (33.5-54.1)
Thoracic spine	36.3 (25.7-46.8)	37.5 (26.9-48.1)	42.7 (32.4-53.0)	43.8 (33.5-54.1)
Lumbar spine	30.0 (20.0-40.0)	37.5 (26.9-48.1)	42.7 (32.4-53.0)	46.1 (35.7-56.4)
	Interval of 28±2 days			
	Boys (n=142)		Girls (n=147)	
	Test % (95%CI)	Retest % (95%CI)	Test % (95%CI)	Retest % (95%CI)
Cervical spine	26.1 (18.8-33.3)	24.6 (17.6-31.7)	38.8 (30.9-46.7)	44.2 (36.19-52.3)
Thoracic spine	23.2 (16.3-30.2)	26.8 (19.5-34.0)	38.1 (30.2-46.0)	38.8 (30.90-46.7)
Lumbar spine	28.9 (21.4-36.3)	37.3 (29.4-45.3)	41.5 (33.5-49.5)	44.2 (36.19-52.3)

p>0.05 for all comparisons of relative frequencies between test-retest by McNemar’s test; %: relative frequencies of spine pain; 95%CI: 95% confidence interval.

**Table 4:** Agreement with the *Kappa* index and relative frequencies in reported pain according to gender and interval of days between test-retest.

	Interval of 10±3 days			
	Boys (n=80)		Girls (n=89)	
	***Kappa* (95%CI)**	%	***Kappa* (95%CI)**	%
Cervical spine	0.76 (0.60-0.92)	90.1	0.77 (0.64-0.90)	88.8
Thoracic spine	0.71 (0.54-0.87)	86.3	0.75 (0.61-0.89)	87.7
Lumbar spine	0.61 (0.43-0.79)	82.5	0.70 (0.56-0.85)	85.4
	Interval of 28±2 days			
	Boys (n=142)		Girls (n=147)	
	*Kappa* (95%CI)	%	*Kappa* (95%CI)	%
Cervical spine	0.44 (0.27-0.61)	78.9	0.64 (0.51-0.76)	82.4
Thoracic spine	0.61 (0.46-0.76)	85.2	0.43 (0.28-0.58)	72.2
Lumbar spine	0.50 (0.35-0.64)	77.4	0.53 (0.39-0.67)	76.9

p<0.01 for all *Kappa* values; %: relative frequencies; 95%CI: 95% confidence interval.

With the interval of 10 days between test and retest application of the pain scale, all values of ICC were considered excellent (ICC ≥0.77) for boys and girls. When the interval between applications was higher, the reliability for the boys was good and excellent for the cervical, thoracic and lumbar spine regions. Among girls, the test-retest reliability was excellent for the cervical spine and good for the thoracic and lumbar spine. The agreement of pain scores showed that the mean differences were close to 0. The largest mean difference in the 10-day period was observed among boys for the lumbar spine with -0.16 (95%CI -3.17-2.85). For 28 days, the highest average value of the difference was observed for the girls in the lumbar spine, with -0.40 (95%CI -5.14-4.34) (Table 2).

**Figure 2:**
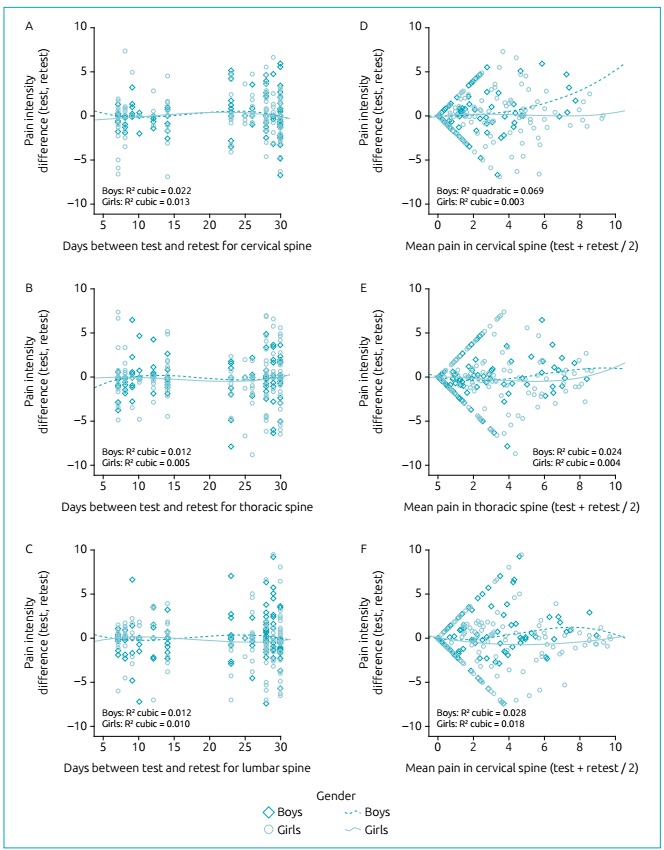
Analysis of the influence of the number of days between test-retest (A, B and C) and average values of pain (D, E and F) on the differences obtained between test-retest.

The number of days between test-retest had a slight influence on the magnitude of the differences. In all cases, the models with the best fits were cubic; the highest variation explained only 2.2% of the differences. These findings suggest that the differences are independent on the number of days between test-retest in this study (Figures 2A, 2B and 2C). The bias for the differences between test-retest and mean values of pain was analyzed considering only the stratification by sex. In general, the models with the best fits were cubic, except for the cervical spine in boys. For this, the best fit was the quadratic model, explaining less than 7% of the variance of the results, and being the highest value obtained (Figures 2D, 2E and 2F).

Table 3 shows that there were no significant differences in frequency of individuals who reported pain in the cervical, thoracic and lumbar spine between test-retest. This fact was evidenced by the McNemar’s test and can also be seen by the overlap of 95%CI in frequencies. The major difference in the frequency of reporting pain in the test-retest interval of 10 days for the boys was found in the lumbar spine with 7.5 percentage points, and for girls in the cervical spine with 4.5 percentage points. In the period from 28 days, the same regions had the greatest variation with a difference of 8.4 percentage points for boys and 5.4 percentage points for girls.

With the exception of the cervical spine for boys (26.1 *vs*. 24.6%) in the interval of 28 days, the frequency of reported pain was slightly higher in the retest moment (Table 3). Generally, higher frequencies of pain were reported among girls in both the test and retest of the instrument for all regions independently on the group of days range. Considering the same interval of days for application, the only region that showed no overlap of 95%CI between gender was the thoracic spine, with the application interval of 28 days between test moments - boys: 23.2% (95%CI 16.29-30.19) *versus* girls: 38.1% (95%CI 30.24-45.95).

The agreement in reported pain (Table 4) for 10 days was considered good for boys and girls. *Kappa* statistics ranged from 0.61 (82.5%) to 0.76 (90.1%) among boys, and 0.70 (85.4%) to 0.77 (88.8%) among girls, according to the examined region. For boys, in the 28 days period between the test-retest, the agreement for the pain scale in both the lumbar spine and cervical spine were moderate [*Kappa* 0.44 (78.9%) and 0.50 (77.4%), respectively], and the thoracic spine was good [*Kappa* 0.61 (85.2%)]. For the girls, in this same 28-day interval the agreement was good for the cervical spine [*Kappa* 0.64 (82.4%)] and moderate for the thoracic and lumbar spine [*Kappa* 0.43 (72.2%) and 0.53 (76.9%), respectively].

## DISCUSSION

The main findings of this study were acceptable values of the instrument on test-retest reliability and agreement of pain frequency and pain intensity independently on day intervals. It should be considered that it was not expected to find perfect reproducibility. Aspects such as memory, seasonality of the investigated phenomenon or the clinical condition of the participant on the assessment of day might influence the obtained information. In the present study, it was found that the differences between the test-retest were not affected by the number of days. Such information may be of great interest when the instrument is used for multiple measurements.

To identify the reproducibility of an instrument proposed to verify the pain, it is a presumption to start using such method. However, some methodological considerations should be done regarding studies that verified back pain in Brazilian young people.^2^ ^,^ ^3^ ^,^ ^4^ ^,^ ^10^ ^,^ ^11^ ^,^ ^21^ The Nordic musculoskeletal questionnaire has been widely used to analyze back pain among Brazilians,^3^ ^,^ ^4^ ^,^ ^10^ ^,^ ^11^ Despite the fact that it has a Portuguese version,^22^ the reproducibility of this Nordic questionnaire was not tested in Brazilian youths.^3^ ^,^ ^4^ ^,^ ^10^ ^,^ ^11^ Dorneles et al.^2^ used a questionnaire that has no Portuguese version, and the reliability data was not described neither for original instrument nor for the study conducted.^23^ Also, in studies that reproducibility was analyzed, the interval of test-retest assessment is usually seven days.^15^ ^,^ ^21^ Therefore, it is not possible to know whether the instruments are reproducible in larger time intervals. The scale proposed in the present study showed reproducibility during a period of more than 10 days, providing support for the use of the spine pain instrument.

Although the instruments described before provides valuable information for observational studies, such as prevalence of musculoskeletal pain, only categorical outcome (*e.g.*, presence *vs*. absence and frequency of pain) limits the utilization of these scales in intervention studies. In experimental studies that investigated interventions for back pain treatment, it is necessary to check how procedures can reduce pain intensity.^24^ ^,^ ^25^ Noll et al. partially reduced this limitation and proposed an instrument that, in addition to closed questions, had visual analogue scale (0 to 10) to estimate pain intensity.^15^ However, pain intensity is assessed considering general back pain and do not specifies region. In the present study, the instrument was developed to assess presence and intensity of pain using a visual analogue scale on three regions: cervical, thoracic and lumbar regions. A human body draw has been previously used in studies and it is suggested as an ideal method to identify body regions.^26^ ^,^ ^27^ ^,^ ^28^ These characteristics, such as simplicity and possibility of identifying the pain region, contribute to instrument applicability in epidemiological and experimental studies.

The main limitation of the present study is the fact that the validity of the scale was not described. In young people, the criterion validity process of pain scales is conducted matching the results to their clinical diagnose records or to secondary outcomes (*i.e.*, disability).^29^ ^,^ ^30^ Unfortunately, no information about clinical records of the sample was analyzed. Other limitation of this study involved the fact that it was not a population-based survey, with a representative sample. Despite the limitation, the scale is recommended, as it is self-administered, easy to use and understand, as well as it has low cost, being suitable to use in epidemiological studies.

The results of this study support the possibility of using this instrument to screen Brazilian adolescents with spinal pain, and to supply an indicator of the intensity of the pain. It also enables the diagnosis of possible factors associated with the presence of spinal pain or analysis of the effects of intervention to reduce spinal pain. Future studies are suggested to verify the accuracy of the scale in diagnosing cervical, thoracic and lumbar spinal pain in adolescents when compared with a clinical examination and the relationship between spinal pain and postural deviations (examined by imaging methods such as X-rays), spinal injuries and bad posture habits.

In conclusion, the proposed instrument is a reliable tool to verify both presence and intensity of pain in different regions of the spine in Brazilian young people.
